# Consensus-Expressed CXCL8 and MMP9 Identified by Meta-Analyzed Perineural Invasion Gene Signature in Gastric Cancer Microarray Data

**DOI:** 10.3389/fgene.2019.00851

**Published:** 2019-09-25

**Authors:** Xiuzhi Jia, Minjia Lu, Chen Rui, Ying Xiao

**Affiliations:** ^1^Central Lab of Biomedical Research Center, Sir Run Run Shaw Hospital, School of Medicine, Zhejiang University, Hangzhou, Zhejiang Province, China; ^2^Research Department, Hangzhou Beiwo Meditech Co., Ltd, Hangzhou, Zhejiang Province, China

**Keywords:** the cancer genome atlas, gene expression omnibus, fixed-effects model, random-effects model, gene expression profiling interactive analysis

## Abstract

As an underrecognized route of cancer metastasis, perineural invasion (PNI) is defined as the neoplastic invasion of nerves, which can be targeted to inhibit the metastasis of malignant cancer. However, the mechanism underlying PNI in cancer is largely unknown. We constructed a PNI gene signature based on a Pathway Studio–mediated literature screen and investigated the relevant genes in a gastric cancer model. Thus, a total of 467 studies/datasets were retrieved from the Gene Expression Omnibus database using the keyword “gastric cancer,” among which 13 studies that focused on gene expression profiling were further manually inspected and selected. Furthermore, the constructed PNI gene signature (104 genes) expression was meta-analyzed, and the consensus-expressed C-X-C motif chemokine ligand 8 (CXCL8) and matrix metallopeptidase 9 (MMP9) (*p* < 0.01, |log fold change| >1) were detected. Importantly, the disease-free survival was significantly worse in patients with high expressions of CXCL8 and MMP9 than in those with low expressions (*p* = 0.05). Moreover, multiple linear regression analysis showed that the population region (country) was associated with the expressions of both CXCL8 and MMP9. In conclusion, these data suggest that the coexpression of CXCL8 and MMP9 could be an early detection marker for PNI, with a potential to be utilized as individual therapy targets for early treatment to prevent PNI-related cancer metastasis.

## Introduction

As a worldwide health concern owing to its metastasis and recurrence, gastric cancer is one of the most common malignant neoplasms and the second leading cause of cancer-relevant deaths (Sawaki et al., 2018; Khan and Shah, 2019; Necula et al., 2019). In China, patients are often diagnosed in the late stage, and the 5-year overall survival is less than 50% even after radical resection; most of the patients die of recurrence and metastasis (Riihimaki et al., 2016; Zong et al., 2016). It is worth noting that metastatic gastric cancer is a very serious and quickly spreading malignancy, which usually begins in the innermost mucus lining of the stomach, quickly invades deeper layers of muscle tissue, and metastasizes to the abdominal cavity, esophagus, intestines, liver, pancreas, and lymph nodes within several months. To reduce the risk of widespread metastasis and potentially fatal complications, early diagnosis and treatment are urgently recommended.

For some tumors, perineural invasion (PNI) could be detected without vascular or lymphatic invasion and may be the sole route for the metastatic spread, which can cause local recurrence and distant metastasis (Liebig et al., 2009; Liu et al., 2012; Arese et al., 2018; Chen et al., 2019). Extensive prospective studies have shown that PNI is an independent prognostic factor affecting the outcome and survival of gastric cancer patients with curative resection, which is independent of the depth of invasion, tumor size, and lymph node status (Deng et al., 2014; Espana-Ferrufino et al., 2018). Thus, early detection of PNI might benefit the early diagnosis of cancer metastasis and treatment for malignant tumor.

Despite the fact that a few detailed mechanisms are deciphered for PNI, little progress has been made to target PNI-related bioprocesses owing to the lack of proper methodologies. Gene expression profiling represents a high-throughput method of screening potential biomarkers and therapeutic targets to better understand PNI. In this study, the constructed PNI gene signature expression in gastric cancer is meta-analyzed, and the consensus expressions of C-X-C motif chemokine ligand 8 (CXCL8) and matrix metallopeptidase 9 (MMP9) are screened, indicating that a CXCL8/MMP9-relevant PNI process could be targeted to prevent further metastasis.

## Materials and Methods

### PNI Gene Signature Construction

The Pathway Studio database (Bonnet et al., 2009) (www.pathwaystudio.com) was utilized to screen the literature until April 2019, and the interactions containing biological gene–PNI relations were extracted to construct PNI gene signatures, all of which could provide extensive coverage and high-quality scientific evidence to investigate the potential functional genes associated with PNI.

### Gene Annotation Analysis

The PNI gene signature was annotated with the Database for Annotation, Visualization and Integrated Discovery (DAVID; http://david.abcc.ncifcrf.gov/) (Huang et al., 2009a; Huang et al., 2009b) based on gene ontology (GO) terms such as biological process, molecular function, and cellular component, which were then iteratively examined by the Fisher exact test and false discovery rate. The annotated terms were further enriched with GOView (http://www.webgestalt.org/GOView), which could present the enrichment results of multiple GO terms under the GO Directed Acyclic Graph (DAG) structure (Wang et al., 2017).

### Gene Expression Data Retrieved From Gene Expression Omnibus

A systematic search was conducted with expression datasets from Gene Expression Omnibus (GEO, https://www.ncbi.nlm.nih.gov/geo/) (Clough and Barrett, 2016). In total, 467 datasets/series were identified on the basis of the keyword “gastric cancer.” Thirteen of these 467 studies, which satisfied the selection criteria, were included in the meta-analysis, as shown in **Table 1**. As indicated in **Table 1**, expression data detected in different platforms were combined in the GSE37023 dataset, so the data analyzed in the different platforms were analyzed separately. The selection criteria were as follows: (1) the data type was expression profiling by array; (2) the overall design was gastric cancer case versus healthy control; (3) the sample size was greater than or equal to 20; (4) both the number of cases and control groups were greater than or equal to 5; and (5) the sample organism was *Homo sapiens*.

**Table 1:** Gastric cancer dataset employed for meta-analysis.

GEO ID	Contributor	No. Controls	No. cases	Country	Sample organism
GSE19826	Peng et al., 2010	15	12	China	Homo sapiens
GSE3438	Kim et al., 2006	49	50	South Korea	Homo sapiens
GSE13861	Cho et al., 2011	19	65	USA	Homo sapiens
GSE13911	De Rinaldis et al., 2008	31	38	Italy	Homo sapiens
GSE29272	Wang et al., 2013	134	134	USA	Homo sapiens
GSE29998	Holbrook et al., 2012	49	50	Singapore	Homo sapiens
GSE31811	Kitamura et al., 2014	17	21	Japan	Homo sapiens
GSE37023	Wu et al., 2012 (GPL97)	36	29	Singapore	Homo sapiens
GSE37023	Wu et al., 2012 (GPL96)	36	112	Singapore	Homo sapiens
GSE44740	Varro et al., 2013	12	12	United Kingdom	Homo sapiens
GSE64951	Yoshizawa et al., 2015	31	63	USA	Homo sapiens
GSE79973	Shao et al., 2016	10	10	China	Homo sapiens
GSE81948	Sacconi et al., 2017	5	15	Italy	Homo sapiens

### Meta-Analysis Models

Both the fixed-effects model and random-effects model were utilized to study the effect size of the PNI gene signature in the cases of gastric cancer. The expression data were normalized and log2-transformed if these were not done in the original dataset. For each expression dataset, the log fold change (LFC) was calculated and used as the index of effect size in the meta-analysis. The heterogeneity of the meta-analysis was analyzed to indicate the variance within and between different studies. If the total variance *Q* was equal to or smaller than the expected between-study variance df, the statistic ISq = 100% × (*Q* − df)/*Q* would be set as 0, and a fixed-effects model was selected for the meta-analysis. Otherwise, a random-effects model was selected. The *Q*-*p* value represented the probability that the total variance was derived from the within-study variance only.

Significant genes from this meta-analysis were reported, which were identified with the following criteria: *p* < 0.01 and effect size (LFC) >1 or <−1. When a gene presented an effect size (LFC) >1 or <−1 in the meta-analysis, it meant that the change of the expression was greater than twofold or smaller than 0.5-fold.

### Gene Expression Profiling Interactive Analysis

As an interactive website based on The Cancer Genome Atlas (TCGA) project data, Gene Expression Profiling Interactive Analysis (GEPIA) RNA-seq data (http://gepia.cancerpku.cn/) (Tang et al., 2017) were extracted to obtain CXCL8 and MMP9 expressions in human stomach adenocarcinoma and adjacent healthy tissues. The Pearson correlation test was utilized to determine the relationship between the CXCL8 and MMP9 expressions, as indicated by transcripts per kilobase million. Moreover, GEPIA data were utilized to determine the effects of CXCL8 and MMP9 coexpression on disease-free survival.

### Multiple Linear Regression Analysis

A multiple linear regression (MLR) analysis was employed to study the possible effects of sample size, population region, and study date on the gene expression change in disease. *P* values and 95% confidence intervals were reported for each of the factors. This analysis was carried out in Matrix Laboratory (MATLAB, R 2017a) with the “regress” statistical analysis package.

## Results

### Functional Annotation Analysis of PNI Gene Signature

**Figure 1:**
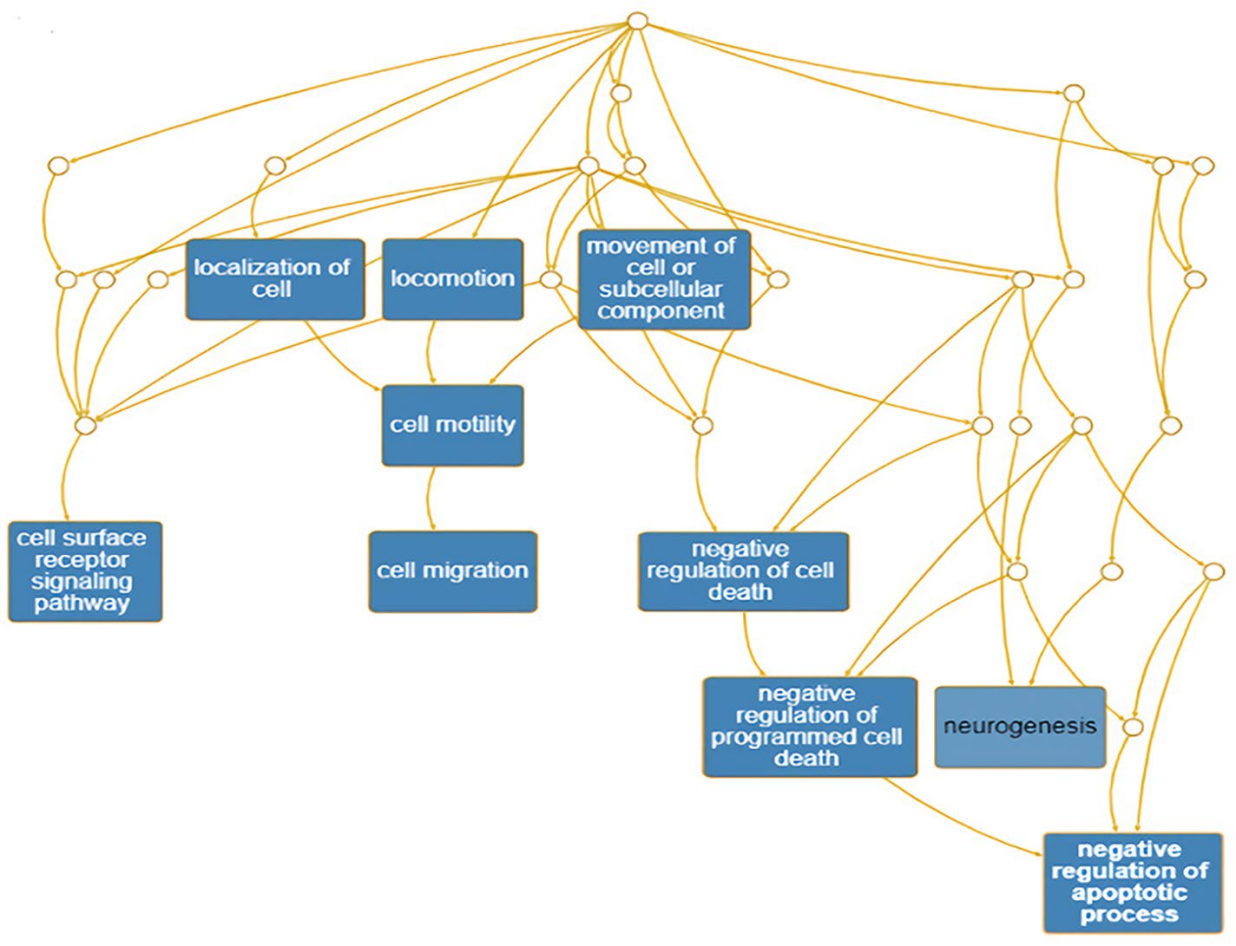
Enrichment of annotated GO terms under the GO Directed Acyclic Graph with PNI-related genes.

The PNI gene signature was obtained and constructed on the basis of Pathway Studio analysis, and a total of 104 genes were found to be attributed to the PNI gene signature (**Supplemental Table 1**). The PNI gene signature was annotated with DAVID, and the annotated GO terms were further enriched and presented with interactive DAG visualization. As depicted in **Figure 1**, most of the enriched GO categories identified for the PNI gene signature were closely related to the function of “cell surface receptor signaling pathway,” “cell migration,” “negative regulation of apoptotic process,” and “neurogenesis,” which are the main bioprocesses involved in PNI. This analysis gave clues that the molecular pathways that participate in the mechanism of tumor cell migration and survival could be responsible for the initiation of PNI.

### Differential Expressions of CXCL8 and MMP9

**Figure 2:**
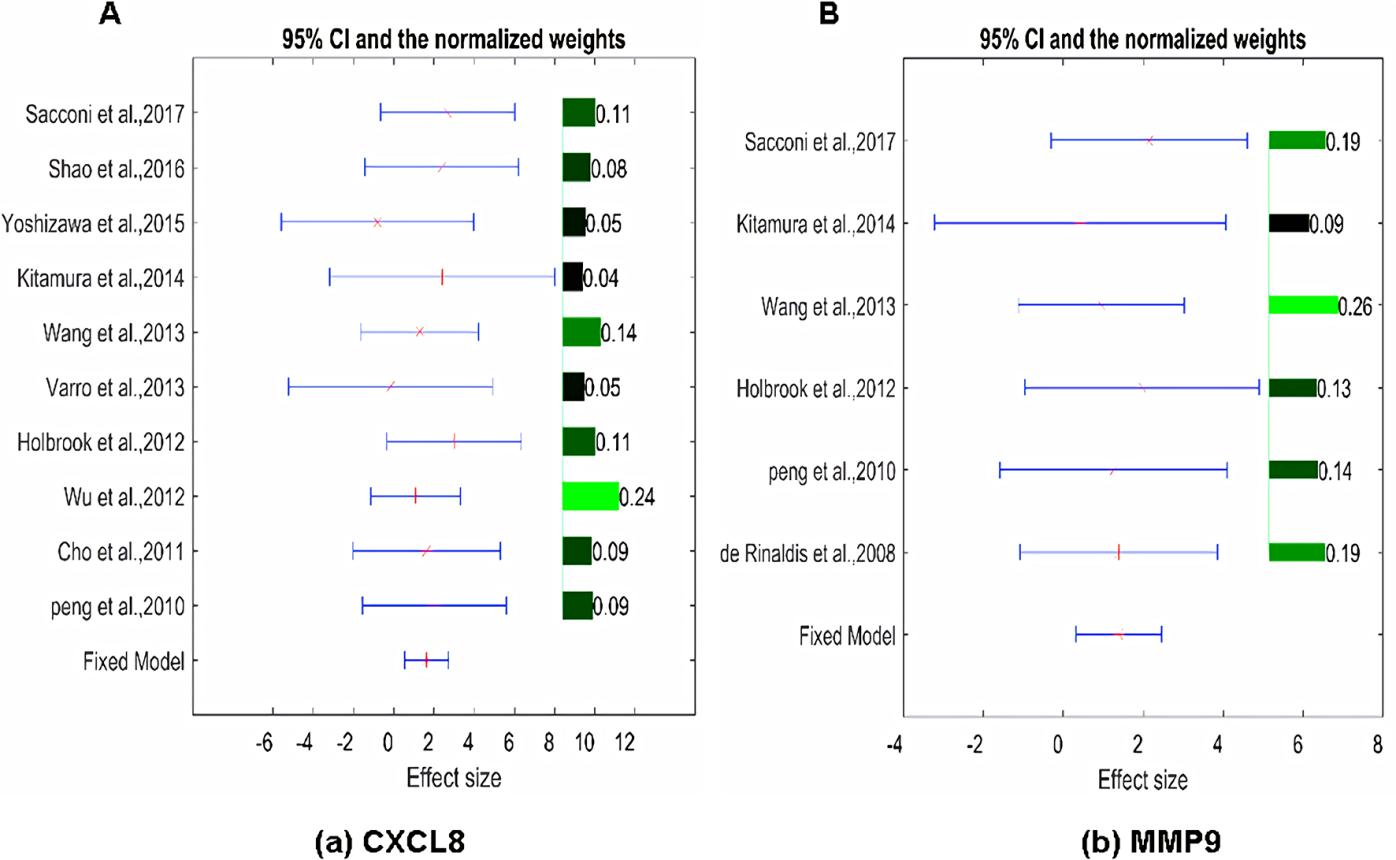
Effect size, 95% confidence interval, and weights for the genes **(A)** CXCL8 and **(B)** MMP9 from the meta-analysis results. The bar plot on the right of each figure represents the normalized weights for each dataset/study, which ranged between 0 and 1; the brighter (green) the color, the larger the weight (labeled right next to the bar). The star (in red) and lines (in blue) on the left are the mean of effect size (log fold change) and 95% confidence interval (CI) of each dataset/study, respectively.

**Figure 3:**
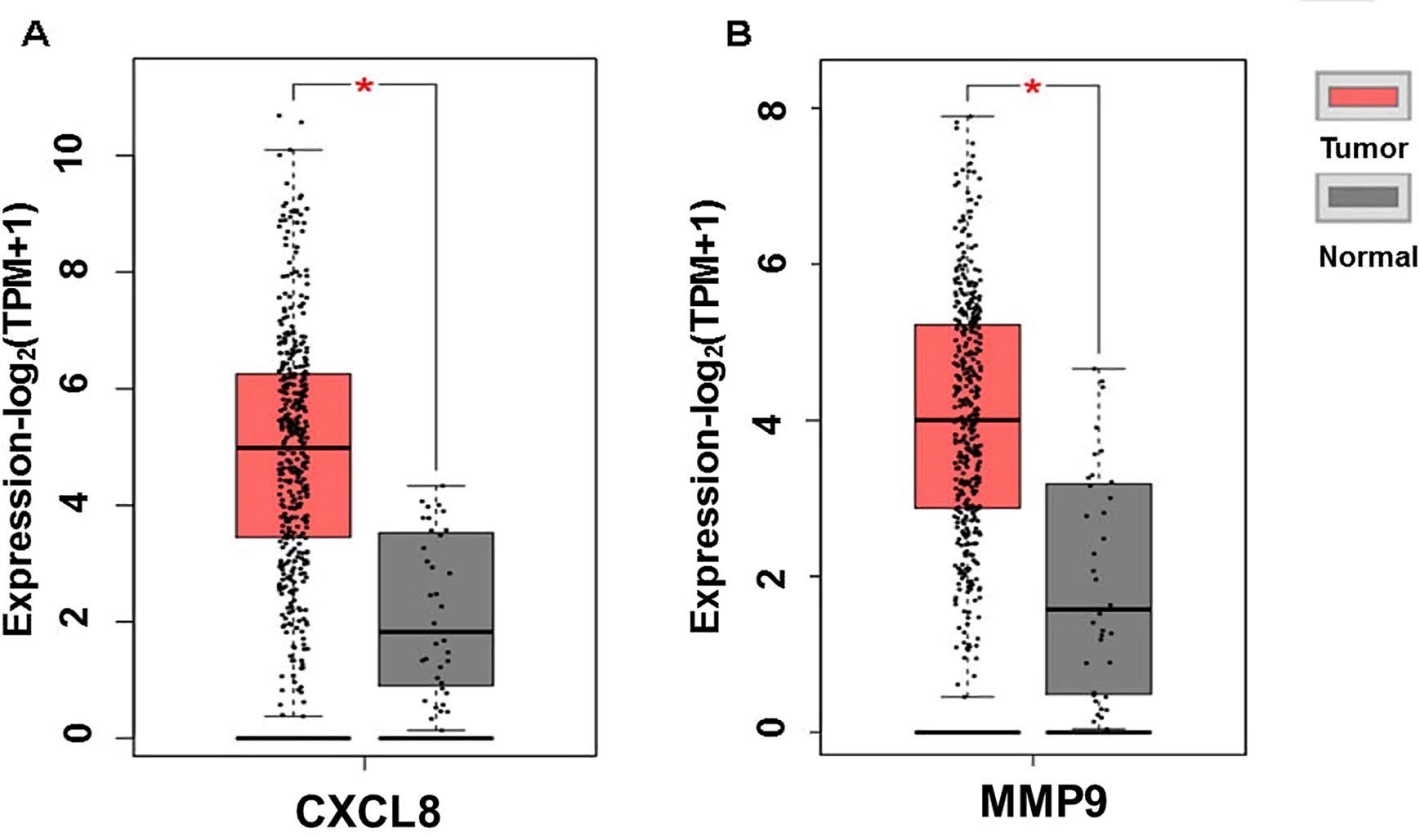
Plot of differential expressions of CXCL8 **(A)** and MMP9 **(B)** in human stomach adenocarcinoma (STAD) tissue (n = 408) compared with normal tissue (n = 32). *p < 0.05.

**Figure 4:**
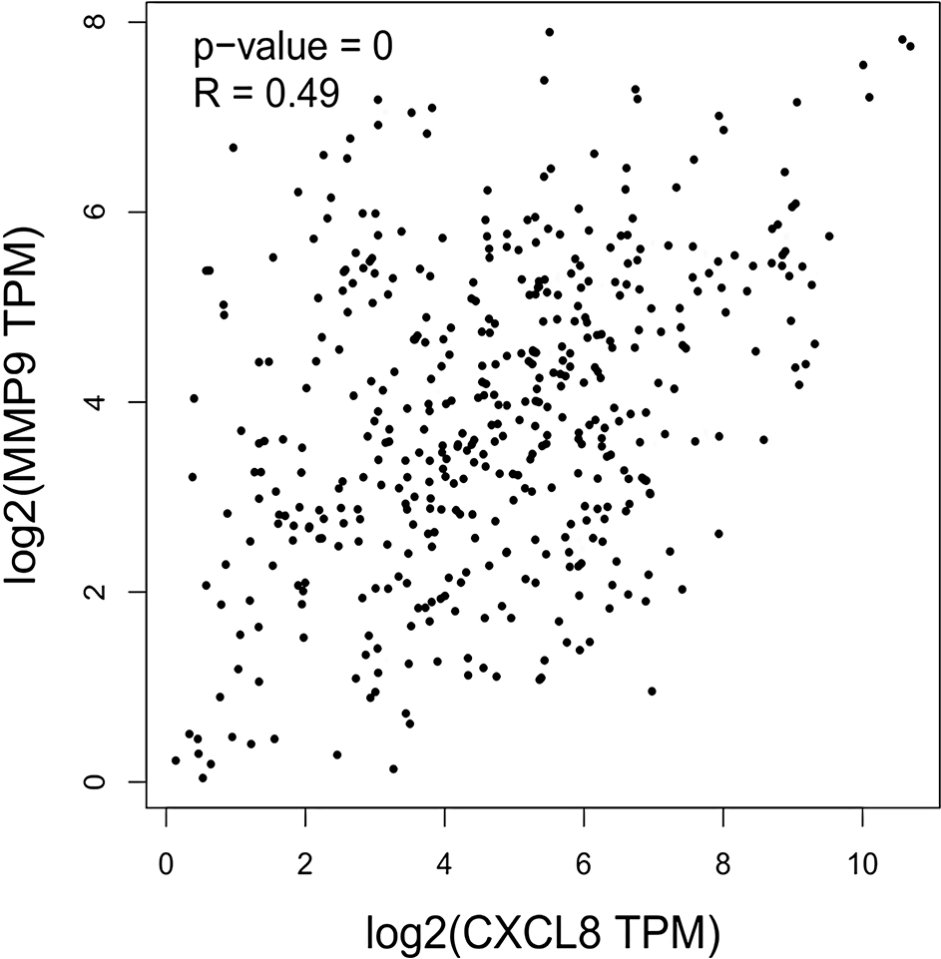
Correlation between CXCL8 and MMP9 expressions as indicated by transcripts per kilobase million (TPM) among human stomach adenocarcinoma (STAD) patients.

**Figure 5:**
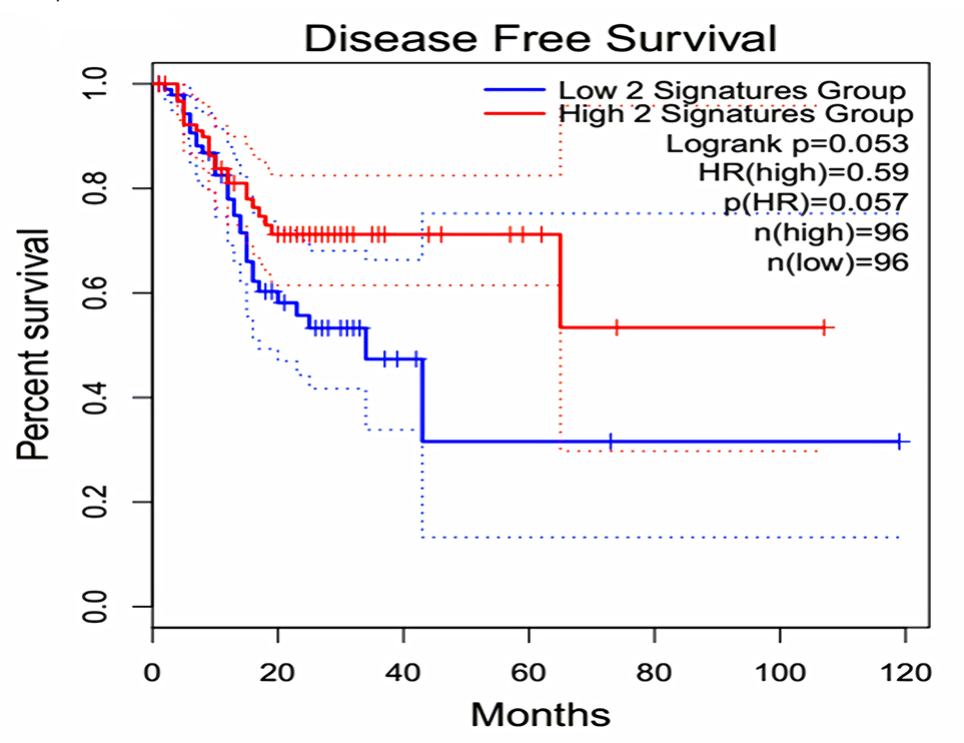
Effect of coexpression of CXCL8 and MMP9 on disease-free survival among gastric cancer patients. Log-rank test was performed to indicate the disease-free survival analysis based on CXCL8 and MMP9 gene-pair expressions. The Cox proportional hazard ratio (HR) and 95% confidence interval information indicated by the dotted line can be referred in the survival plot.

The expression of the PNI gene signature in 13 GEO datasets of gastric cancer was meta-analyzed, among which CXCL8 and MMP9 were significantly expressed (*p* < 0.01, LFC >1 or <−1). Heterogeneity analysis showed that there was no significant between-study variance for both CXCL8 and MMP9 (ISq = 0, *p*-*Q* = 0.96), and therefore, a fixed-effects model was selected. The effect sizes and relevant statistical parameters are presented in **Table 2**, and the 95% confidence interval and weights of each study are presented in **Figure 2**. The higher expression levels of both CXCL8 and MMP9 in a gastric cancer sample were also confirmed in the TCGA data when compared with a normal sample (**Figure 3**) analyzed with GEPIA (Tang et al., 2017), and it was further found that the correlation between CXCL8 and MMP9 expressions was *R* = 0.49 (**Figure 4**, *p* = 0). Importantly, the relative expressions of CXCL8 and MMP9 signatures (high or low coexpression) could significantly affect disease-free survival (**Figure 5**, *p* = 0.05). All these indicated that the CXCL8 and MMP9 coactivated bioprocess might be the consistent phenotype involved in PNI-related gastric cancer.

**Table 2:** Significant genes identified from meta-analysis.

Gene name	Random-effects model	No. studies	LFC	p	ISq (%)	p-Q
CXCL8	NO	10	1.64	1.70e−3	0	0.96
MMP9	NO	6	1.40	5.11e−3	0	0.96

p Value represents the probability that the fold change is equal to 0. ISq = 100% × (Q − df)/Q represents the percentage of between-variance over total variance; p-Q represents the probability that the variance is coming from within-study only. LFC, log fold change (the effect size).

### Population Region Contribution to the Differential Expressions of CXCL8 <br/>and MMP9

Multiple linear regression analysis showed that population region (country) was a significant factor influencing the expressions (fold change) of both CXCL8 and MMP9 (*p* = 1.44e−3 and 2.12e−4, respectively) as revealed in **Table 3** and **Supplemental Figure 1**, whereas sample size and study date did not show such effect. Although large prospective studies are needed to confirm such observation, the population-correlated expression pattern indicated the necessity of considering a population-based treatment strategy.

**Table 3:** Multiple linear regression analysis on sample size, population region, and study date.

Gene name	Sample size	Population region	Study date
CXCL8	0.30	1.44e−3	0.074
MMP9	0.53	2.12e−4	0.99

## Discussion

Perineural invasion is considered to be one of the determining factors that affect local recurrence and metastasis after resection. For the first time, a PNI gene signature (104 genes) is constructed in this investigation by the text mining method. The consensus-expressed PNI gene signature (CXCL8 and MMP9) in gastric cancer is identified on the basis of meta-analyzed GEO datasets. Moreover, the expressions of CXCL8 and MMP9 are associated with population or country, and the coexpression of CXCL8 and MMP9 has an effect on patient survival. A high accident rate of aggressive prostate cancer has been reported in patients of African descent, which can be attributed to the population, presence of PNI, and higher Gleason scores of cancer (Nzioka et al., 2014). All these indicate that individual and racial-dependent therapies based on CXCL8 and MMP9 for early treatment can be potentially utilized to prevent PNI. In summary, such analysis framework can be utilized to decipher molecular targets that can be attributed to a certain bioprocess in PNI.

As a multifunctional proinflammatory chemokine, CXCL8 is significantly upregulated in both the tumor and tumor-derived microenvironment and acts as a key regulator of proliferation, migration, angiogenesis, metastasis, and resistance to chemotherapeutics through binding with the high-affinity CXCR1 and CXCR2 G-protein–coupled receptors (Lee et al., 2013). CXCR2 is upregulated in dorsal horn neurons after spinal nerve ligation, traumatic brain injury, and inflammation stimulation, which contribute to the maintenance of pain (Zhang et al., 2013; Cao et al., 2016; Liang et al., 2017). CXCL8 could stimulate the production and release of MMP2 and MMP9 (Sparmann and Bar-Sagi, 2004; Mantovani et al., 2008), which suggests that the invasiveness and extracellular matrix remodeling process can be modulated in the presence of CXCL8. It is worth noting that CXCL8 is involved in directing organ-specific metastasis to regional lymph nodes and perineurons where CXCR2 is expressed.

CXCL8 and MMP9 overexpression correlates with the poor prognosis of bladder cancer. High-grade tumors express significantly higher levels of MMP9 and CXCL8 than low-grade tumors (Reis et al., 2012). Furthermore, CXCR2 stimulation could promote bladder cancer cell migration and invasion by activating the PI3K/AKT-induced upregulation of MMP2/MMP9 (Gao et al., 2015). It is interesting to note that CXCR2 silencing could reduce the expression of MMP9 and phosphorylated Akt, which indicates that Akt-induced MMP9 expression and activation may be a canonical pathway related to CXCR2 (Wu et al., 2016). In this research, consensus-expressed CXCL8 and MMP9 are identified in gastric cancer, and a higher coexpression can predict poor disease-free survival. All these hint that CXCL8 may mediate the PNI and the relevant abdominal pain through binding with CXCR2 to further activate MMP9, which need further investigation.

High levels of CXCL8 expression in tissue might correlate with the tumorigenicity, angiogenesis, and metastasis of tumors in numerous xenograft and orthotopic models (Waugh and Wilson, 2008). In humans, a high expression level in a tumor sample may be associated with poor prognosis as stratified by tumor stage and pathology classification (Eck et al., 2003; Li et al., 2015). Moreover, it is further shown that high levels of CXCL8 in serum are correlated with disease aggressiveness and an unfavorable initial response to chemotherapeutic drugs, such as camptothecin, 5-fluorouracil, oxaliplatin, and paclitaxel (Lee et al., 2007; Baj-Krzyworzeka et al., 2016). All these indicate that CXCL8 can be utilized as a prognostic and predictive cancer biomarker to indicate a more aggressive phenotype. Therefore, CXCL8 inhibition might be a novel therapeutic strategy in targeting the tumor and the associated microenvironment.

It must be mentioned that CXCL8 can regulate the development and progression of gastric cancer in an inflammatory-chemotaxis–independent manner, although the CXCL8 target fails to explain the signaling effects of other CXC chemokines that emerged in some preclinical trials (Lee et al., 2013). Therefore, a targeted strategy based on receptor selectivity, such as the inhibition of CXCR2, is put forward to eliminate the redundant function of CXCL8 signaling. The present investigation also shows that the expressions of both CXCL8 and MMP9 are related to population region or country, and the coexpression patterns of CXCL8 and MMP9 will affect disease-free survival. All these indicate the necessity of individual and racial-dependent therapies to target the activated CXCL8/MMP9 pathway or PNI bioprocess-specific target strategy to prevent further PNI-related metastasis.

## Conclusions

In this investigation, a novel analytical framework of gene signature based on gene expression profile meta-analysis is proposed. The consensus expressions of CXCL8 and MMP9 might be considered as novel PNI-related targets to define biomarker-based subgroup stratification and to prevent PNI-mediated early metastasis.

## Data Availability

The datasets analyzed for this study can be found in the GEO datasets: GSE19826, GSE3438, GSE13861, GSE13911, GSE29272, GSE29998, GSE31811, GSE37023, GSE37023, GSE44740, GSE64951, GSE79973, GSE81948.

## Author Contributions

XJ and YX designed the investigation. XJ, ML, and CR performed the bioinformatics and statistical analyses. XJ and ML drafted the manuscript. YX revised the manuscript critically. All authors reviewed and approved the final version of the manuscript.

## Funding

This project was supported by the scientific research start-up funds for specially engaged employee of Sir Run Run Shaw Hospital (Ytp1902), the National Nature Science Foundation of China (81660708), and the Key Project of the Tibetan Medical Administration of Tibet (2017005).

## Conflict of Interest Statement

Author ML was employed by company Hangzhou Beiwo Meditech Co., Ltd.

The remaining authors declare that the research was conducted in the absence of any commercial or financial relationships that could be construed as a potential conflict of interest.

## Supplementary Material

Supplemental Figure 1 Population region (country) is a significant factor influencing the expressions (fold change) of both CXCL8 **(A)** and MMP9 **(B)** analyzed by multiple linear regression (MLR). Linear regression rate, LRR.
